# Preoperative Radiation Therapy Followed by Reexcision May Improve Local Control and Progression-Free Survival in Unplanned Excisions of Soft Tissue Sarcomas of the Extremity and Chest-Wall

**DOI:** 10.1155/2016/5963167

**Published:** 2016-10-10

**Authors:** Hina Saeed, David M. King, Candice A. Johnstone, John A. Charlson, Donald A. Hackbarth, John C. Neilson, Manpreet Bedi

**Affiliations:** ^1^Department of Radiation Oncology, Medical College of Wisconsin, 9200 West Wisconsin Ave, Milwaukee, WI 53226, USA; ^2^Department of Orthopaedic Surgery, Medical College of Wisconsin, 9200 West Wisconsin Ave, Milwaukee, WI 53226, USA; ^3^Department of Medical Oncology, Medical College of Wisconsin, 9200 West Wisconsin Ave, Milwaukee, WI 53226, USA

## Abstract

*Background*. The management for unplanned excision (UE) of soft tissue sarcomas (STS) has not been established. In this study, we compare outcomes of UE versus planned excision (PE) and determine an optimal treatment for UE in STS.* Methods*. From 2000 to 2014 a review was performed on all patients treated with localized STS. Clinical outcomes including local recurrence-free survival (LRFS), progression-free survival (PFS), and overall survival (OS) were evaluated using the Kaplan-Meier estimate. Univariate (UVA) and multivariate (MVA) analyses were performed to determine prognostic variables. For MVA, Cox proportional hazards model was used.* Results*. 245 patients were included in the analysis. 14% underwent UE. Median follow-up was 2.8 years. The LR rate was 8.6%. The LR rate in UE was 35% versus 4.2% in PE patients (*p* < 0.0001). 2-year PFS in UE versus PE patients was 4.2 years and 9.3 years, respectively (*p* = 0.08). Preoperative radiation (RT) (*p* = 0.01) and use of any RT for UE (*p* = 0.003) led to improved PFS. On MVA, preoperative RT (*p* = 0.04) and performance status (*p* = 0.01) led to improved PFS.* Conclusions*. UEs led to decreased LC and PFS versus PE in patients with STS. The use of preoperative RT followed by reexcision improved LC and PFS in patients who had UE of their STS.

## 1. Introduction

Soft tissue sarcomas (STS) are heterogenous rare malignancies that vary in the way they present and behave unpredictably.

The mainstay of treatment of localized stage I-III STS of the extremity and chest-wall is wide local excision with radiation with or without chemotherapy. Alternatively, low-grade, small (<5 cm), subcutaneous tumors may be treated with wide local excision alone. With these methods of treatment, local control rates are excellent [1–3].

Guidelines from the National Comprehensive Cancer Network (NCCN) indicate that management of STS requires appropriate workup which includes localized and systemic imaging, carefully planned core needle or incisional biopsy, and discussion in a multidisciplinary setting [4].

Due to the rare nature of this disease process and the higher propensity for patients to present with benign masses, there are a considerable number of patients with STS of the extremity and chest-wall who do not undergo the appropriate workup and instead undergo nononcologic or unplanned excisions (UEs), especially by nonsurgical oncologists in the community [5]. It is estimated that up to or over 50% of patients with STS undergo UE and of these approximately 20% to 50% are later referred to a tertiary center for further oncologic management [6–8]. UEs can vary considerably and may leave gross residual disease, microscopic residuum, or less commonly be performed with close but negative margins. The pathology report may provide clues regarding the possibility of residual disease, but the specimens are often evaluated by pathologists unfamiliar with the proper processing of sarcoma specimens. Tumors removed piecemeal and specimens with positive margins are clearly at risk for local recurrence. Contrary to UE, appropriate oncologic resections or planned excisions (PE) involve dissection in normal tissue planes outside of the tumor pseudocapsule with careful attention to minimize the risk of tumor contamination. For instance, even with a negative margin resection, if local anesthetic was utilized prior to excision, the risk of tumor spread into surrounding tissues from the needle tracks exists.

The appropriate management strategy after UEs is challenging and minimal guidance is provided in the existing literature. Management decisions may be impacted by a number of factors including, but not limited to, an analysis of preoperative imaging, histology, margin status, method of excision, surgeon experience, and sarcoma location. Management options include (1) observation, (2) wide reexcision alone, (3) preoperative radiation (RT) followed by wide reexcision, (4) wide reexcision followed by adjuvant RT, and (5) RT alone. Due to the plethora of treatment options and number of complex, interrelated variables that may impact outcomes, treatment recommendations should evolve and emerge from the discussion by a team of experts at a multidisciplinary tumor board. Regardless of additional treatments, disease control after UE is compromised, with inferior outcomes, compared to patients who undergo PE by a sarcoma specialist. The optimal management of patients who undergo UE for their STS remains to be determined.

The goal of this study was to compare outcomes of patients who underwent PE versus UE and to assess if a particular treatment strategy after UE resulted in outcomes comparable to those patients who had a PE.

## 2. Materials and Methods

This research was reviewed and approved by the Medical College of Wisconsin Institutional Review Board (IRB) and all investigators completed training in both human research and patient privacy.

### 2.1. Patient Population

All patients with primary soft tissue sarcomas (STS) of the upper extremity, lower extremity, and chest-wall were retrospectively reviewed between 2000 and 2014. Patient, tumor, and treatment characteristics are located in Table 1. Exclusion criteria included STS of locations other than the extremity or superficial trunk, metastatic disease, histopathologic types of rhabdomyosarcoma, extraosseous primitive neuroectodermal tumor, Ewing's sarcoma, osteosarcoma, Kaposi's sarcoma, angiosarcoma, aggressive fibromatosis, and follow-up less than 6 months. All pathology specimens were reviewed by a fellowship trained musculoskeletal pathologist.

Patients were divided into 2 groups for analysis. The PE group consisted of STS patients who underwent resection after appropriate imaging with MRI or CT of the affected site, systemic imaging if warranted, biopsy, and discussion in a multidisciplinary setting, as well as wide resection by a trained sarcoma surgeon. The UE group consisted of patients who were referred from outside institutions after nononcologic resections were performed. UE was defined as an excisional biopsy or unplanned resection of a mass without consideration for the need for appropriate width negative margins. All patients were staged according to the 2009 American Joint Committee on Cancer (AJCC) system, seventh edition.

Each patient, no matter if they underwent a PE or UE, was presented at our institutional multidisciplinary tumor board where input from surgical, medical, and radiation oncology, radiology, and pathology was elicited in order to provide a comprehensive treatment plan. Recommendations were based on age, disease burden, histology, margin status, symptoms, comorbidities, perceived tolerance to therapies, social factors, such as support and distance to the treating facility, and patient preferences. These proposals as well as alternatives to treatment were discussed at the multidisciplinary tumor board and then discussed with the patient and their family members to determine a plan for management.

### 2.2. Planned Excision

Patients who underwent PE of their primary tumor were either managed with wide local excision alone, RT with or without chemotherapy followed by wide local excision, or wide local excision followed by postoperative radiation. In the PE cases, RT was utilized in patients with deep, large, or high-grade STS. Dose, fractionation, and type of radiation (3D or intensity-modulated RT) were at the discretion of the radiation oncologists. Consistent with our institutional preferences, radiation, if delivered in conjunction with resection, was often performed in a preoperative setting. Preoperative RT was also used to facilitate resection. Patients who were given preoperative radiation received a median dose of 50 Gy at 2 Gy per fraction. Patients who underwent postoperative radiation received a median dose of 60 Gy at 2 Gy per fraction. The entire incision or biopsy site in addition to 3.5 to 5 cm margins superiorly/inferiorly and 1 to 1.5 cm radially was used with daily image-guided radiation therapy (IGRT). Exact margins were contingent upon initial tumor size and grade of disease.

Chemotherapy was administered to select patients who were typically <70 years of age, with large, high-grade sarcomas. Chemotherapy was delivered prior to radiation therapy in a sequential manner, using adriamycin and ifosfamide for 3 cycles. If tumors progressed through chemotherapy, then the 3rd cycle was abandoned and the patient proceeded to preoperative radiation.

Limb-sparing resection was also performed in all patients after discussion in a multidisciplinary setting. Surgery was performed grossly through normal tissue planes with sacrifice of arteries or veins that were involved by tumor. Preservation of neurovascular structures was performed when possible. The goal of surgery was to achieve negative margins. When the percutaneous biopsy site could be identified, it was excised. Reconstructive plastic surgeons were involved in cases with difficult wound closures at the discretion of the musculoskeletal oncologist.

### 2.3. Unplanned Excision

Recommendations for patients who underwent UE for their STS included reexcision with or without the use of radiation, or chemotherapy. Radiation was recommended in the majority of cases, either alone, “preoperatively” followed by planned reexcision, or “postoperatively” after planned reexcision. Wide reexcision involved the removal of the UE scar which encompassed the prior resection site with an additional 2-3 cm circumferential margin, when feasible.

MRI was obtained prior to reexcision and any area with postsurgical changes was included in the planned reexcision. For superficial tumors the deep fascia was removed as an additional margin.

Radiation was delivered prior to planned reresection to the UE site to a median dose of 50 Gy at 2 Gy per fraction. The median dose of radiation delivered in a postoperative setting was 60 Gy at 2 Gy per fraction. Similar margins and IGRT were used in this cohort, compared to their PE counterpart.

### 2.4. Follow-Up Care

Patients at the institution were staged and monitored with the following protocol.

An MRI of the primary site with gadolinium and a PET/CT scan or a CT of the chest, abdomen, or pelvis was obtained for initial staging. Surveillance included a CT of the chest, abdomen, and pelvis every 4 months for 2 years for intermediate to high-grade lesions followed by every 6 months for years 3–5. For low-grade lesions, patients received either CT of the chest, abdomen, and pelvis every 6 months or a chest X-ray, depending on patient preference, for 5 years.

Local recurrence was evaluated with a baseline MRI with contrast 4–6 months following surgical resection. MRIs of the local site were obtained in coordination with systemic surveillance imaging and an office visit with physical exam every 4–6 months for the first 2 years following resection. MRI and physical exam were then performed every 6 months from year 2 to 5. From year 5 to 10 the patient was given the choice of a yearly MRI or physical exam.

### 2.5. Statistical Analysis

The statistical software MedCalc (Version 15.6; MedCalc Software bvba, Ostend, Belgium) was used for all data analysis. Patient, tumor, and treatment characteristics were determined and clinical outcomes including local recurrence-free survival (LRFS), progression-free survival (PFS), and overall survival (OS) were evaluated using the Kaplan-Meier estimate of the survival function. The log-rank test was used to compare two survival curves. The tests were two-sided. The tests for the proportional hazards assumption were not significant at the 0.05 level. Univariate (UVA) and multivariate (MVA) analyses were performed to determine prognostic variables in correlation with the above survivals. For MVA, the Cox proportional hazards model was used.

## 3. Results

### 3.1. Patient and Tumor Characteristics

Two-hundred and forty-five patients with stage I-III STS were identified in our database with more than 6 months of follow-up. Thirty-four (14%) of these patients were referred to our institution after UE and 211 (86%) of these patients underwent PE after appropriate imaging, biopsy, and treatment discussion in a multidisciplinary setting. Median follow-up was 2.8 years (6–27.6 years). Patient and tumor characteristics are located in Table 1.

Age, gender, and histopathologic type distribution was relatively uniform between these 2 groups. Patients with UE more commonly had stage II disease, smaller tumors, and an upper extremity location and were with intermediate grade. There were also a higher proportion of subcutaneous tumors in the UE group (Table 1).

### 3.2. Treatment

Of the patients who had UE, 15 (44%) underwent preoperative imaging with CT or MRI and no patients (0%) underwent preoperative biopsy.  Table 2 lists the various post-UE interventions and corresponding local recurrence (LR) rates. Of the 34 patients who underwent UE, 25 (73.5%) underwent planned reexcision either alone or in conjunction with radiation therapy. Eighteen (72%) of patients who underwent reexcision had residual disease in the resection specimen. Four (22%) of the patients with residual disease had subcutaneous STS, 13 (72%) had high-grade disease, and 9 (50%) had lower extremity tumors. All patients had negative margins upon reexcision. No patient required an amputation.

Twenty-two patients received radiation and 12 received no radiation therapy. The 22 patients that received radiotherapy received this either after UE alone (5 patients), followed by reexcision (6 patients), or “preoperatively” prior to planned reexcision (11 patients) (Table 2). Of the patients that underwent reexcision after UE, 7 (28%) had no residual disease. Of those that had no residual disease, 4 (57%) had preoperative RT followed by reexcision, 2 (28.7%) had reexcision alone, and 1 (14.3%) had reexcision followed by postoperative RT.

Chemotherapy alone was delivered in 1 patient (2.9%) and observation was done in 8.8% of STS patients who preferred no further management of their disease.

### 3.3. Outcomes

**Figure 1:**
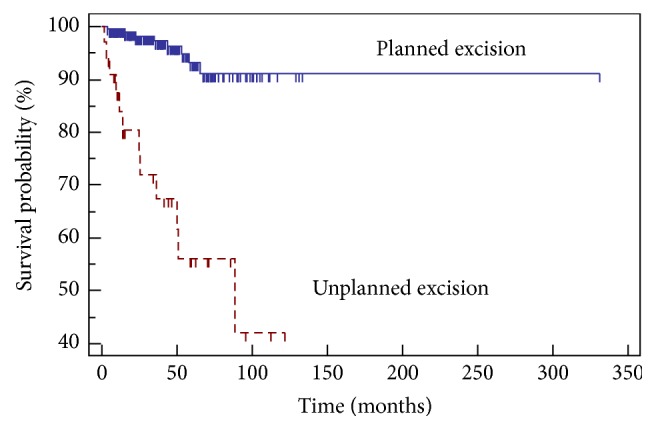
Local recurrence-free survival and excision type.

The median OS and 2-year OS for the entire cohort were 14.8 years and 88%, respectively. The median PFS and 2-year PFS for the entire cohort were 9.3 years and 75%, respectively. The overall local recurrence (LR) rate was 8.6%. Twelve patients in the UE group developed LR. The LR rate for patients undergoing UE was 35% versus 4.2% in those who underwent a PE (*p* < 0.0001) (Figure 1). The median time to recurrence was 19 months in patients undergoing UE and 39 months in patients that underwent PE. In the UE group, 8/12 patients (66.7%) that had a LR had high-grade disease on initial presentation and 8/12 (66.7%) had STS that invaded into the fascia or the muscle (TXb).

The 2-year PFS for patients undergoing UE was 4.2 years versus 9.3 years in patients undergoing a PE (*p* = 0.08). The LR rate for UE patients undergoing preoperative RT followed by reexcision was 9% (1/11), 66.7% (8/12) in those that received no RT (chemotherapy alone, reexcision alone, or observation alone), and 27% (3/11) in those that underwent postoperative RT (reexcision and postoperative RT or post-UE RT). There was no difference in OS between patients who underwent UE versus PE.

**Figure 2:**
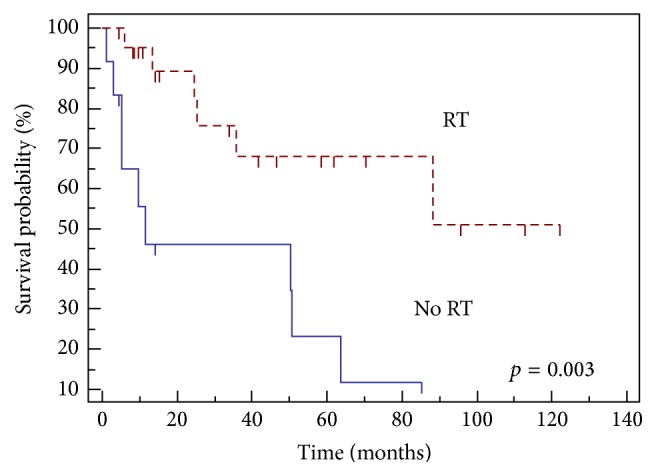
Progression-free survival with and without radiation.

**Figure 3:**
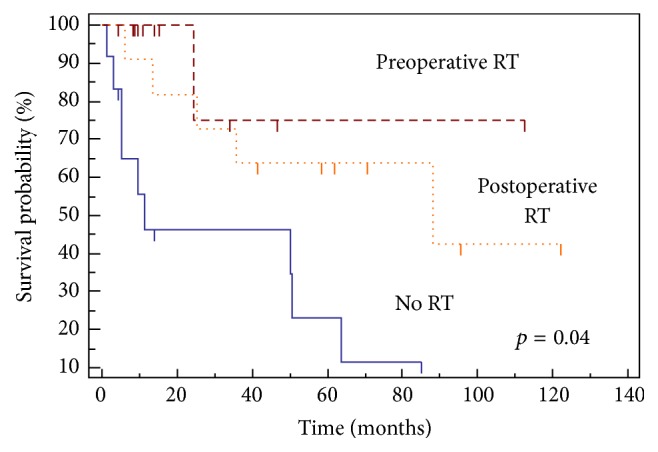
Progression-free survival and timing of radiation.

Significant variables on UVA for LRFS, PFS, and OS for patients undergoing UEs are listed in Table 3. Median PFS for patients who underwent preoperative RT was not met (>120 months) compared to 11.5 months in patients who did not receive RT and 88.1 months in patients who received postoperative RT. Use of radiation therapy (*p* = 0.003) (Figure 2) and administration of preoperative RT (*p* = 0.01) (Figure 3) both led to improved PFS in patients with UE.

On MVA for LRFS, decreased performance status (*p* = 0.01) led to decreased LRFS. On MVA for PFS, preoperative RT (*p* = 0.04) and increased performance status (*p* = 0.01) led to improved PFS. There were no factors that predicted for increased OS on MVA in patients who underwent UE.

## 4. Discussion

STS are rare entities and can be misperceived as a benign process. Thus, nonsurgical oncologists, orthopedic surgeons, and primary care providers are at risk of performing unplanned excisions of these tumors due to a low index of suspicion for potential malignancy [9, 10]. Giuliano and Eilber initially described the term “unplanned total excision” and raised caution regarding the high incidence of gross residual tumor tissue in these cases [11]. The UE of a lesion is further defined as lacking the benefits of preoperative diagnostic modalities and with no intention to achieve tumor-free margins [12, 13].

Unfortunately, several reports have found that the incidence of unplanned resection of STS ranges from 18% to 65% [7, 13–22], suggesting that unplanned resections of soft tissue sarcomas are still frequently performed. Our data shows that 14% of our STS patients from 2000 to 2014 received unplanned excision at an outside facility and all were referred to us for additional management. Although our study reports a lower incidence of UEs compared to other reports in the literature, this may reflect a well-established stable referral base to our tertiary sarcoma center and, hopefully, increased recognition that preoperative imaging is recommended for large, deep masses.

Several studies have shown that patients who undergo unplanned excisions have a substantially higher rate of LR compared to those that undergo planned excisions, from 10% to 38% [15, 16, 19, 20]. This can be potentially explained by contamination of neighboring compartments with malignant cells due to nononcologic resection techniques and without a goal to achieve R0 resection. In this study, the rates of LR were higher in the UE excision group (35% versus 4.2%), which could be due to the low sample size in the UE group of 34. However, this could also be due to seeding of cells due to the lack of appropriate resections and margins achieved.

Nononcologically excised STS are often small and subcutaneous [14, 17, 23–25] which is consistent with the patients in our series. Small, subcutaneous masses are more likely to be mistaken for benign entities such as lipomas and excised by nononcologic surgeons. Conversely, signs suggestive of malignancy such as size > 5 cm, rapid increase in size of the mass, presence of mass deep to fascia, pain in previously painless mass, and recurrent swelling are more alarming and result in a referral to specialized sarcoma centers for proper management of the disease [9, 26].

Due to a higher frequency of benign lumps and lack of tumor and patient characteristics that can be helpful in identifying this subset, unplanned excisions will likely continue. Our study, which is consistent with the literature, confirms worse oncologic outcomes with UE. It is therefore imperative to assess and analyze treatment patterns that result in superior outcomes for an initially UE.

It is well documented that proper diagnostic modalities, preoperative imaging, and presence of multidisciplinary tumor board to implement multimodality therapy in specialized sarcoma centers are essential for excellent outcomes in sarcoma [4, 9, 10, 27]. For every UE case referred to our institution, the treatment plan was based on the recommendations of the tumor board.

Once an UE has been carried out, there is a general consensus for the need for reexcision by an experienced surgical or musculoskeletal oncologist to remove the residual tumor with an appropriate margin [9, 11–13, 15, 17, 18, 28–30]. Previous studies have demonstrated the presence of residual tumor in reresected specimens to range from 24% to 91% [7–9, 11–13, 15–18, 20, 29–34]. Given the high rate of residual disease, the further need for local therapy is undeniable, even without evidence of gross residual disease on postexcision MRI. Manoso et al. reported that MRI carried a false-negative rate of 25%, further suggesting that imaging cannot reliably predict the presence of residual disease [15]. In our study, 72% of reexcised cases had residual disease, in agreement with the literature. Furthermore, no patients had positive margins upon reexcision and none required amputation.

The presence of residual disease in reexcised specimens is associated with worse oncologic outcomes including increased local recurrence and metastasis [9, 19]. Davis et al. reported recurrence rates in 16.6% of patients with residual disease versus 1.6% for patients without residual disease [12]. Other studies have also reported improved RFS, distant metastasis-free survival, and/or overall survival in those without residual disease [17, 20]. Fiore et al. showed that patients with microscopic residual disease had higher incidence of distant metastasis on multivariate analysis [17]. Some authors have suggested that unplanned resection is not a significant risk factor for a worse oncological outcome in terms of local recurrence, metastasis, and overall survival rates; however the validity and reproducibility of these findings have been questioned [14, 17, 35–37].

Those patients without residual tumor also have an increased risk of local recurrence. In the series reported by Chandrasekar et al., 10% of patients with no residual disease upon reexcision developed a local recurrence. Notably, 115 patients in this series without residual tumor or local recurrence had their disease controlled by either excision or adjuvant radiotherapy. Despite reexcision, the risk of local recurrence is significant and is associated with high-grade tumors, residual tumor, marginal reexcisions, and deep tumors [7, 15, 20]. Thus, other factors impact disease control which may necessitate further intervention in these scenarios.

RT may be employed in the setting of UE; however the use and timing are not well established. Postoperative radiotherapy has been employed routinely, either after UE or after reexcision, but has not led to improved outcomes [15, 19]. Potter et al. assessed patients with both PE and UE. In this series, 64% of patients with UEs had radiotherapy. The majority of patients received postoperative RT (116/122). In this study, the use of radiation did not influence local recurrence rates [19]. However, it should be noted that 60% of patients with UE received chemotherapy, as did patients with large, deep tumors and advanced stage disease. In the current study, the LR rate was 58% in those that received no RT and 27% in patients undergoing postoperative radiation.

Although postoperative RT may still afford a relatively high LR rate, preoperative radiation seems to be promising in terms of the management of UE. In our study, 65% of the UE group received radiation therapy, including 32% who underwent preoperative radiation therapy. The LR rate was 9% in those that received preoperative radiation and there was an improvement in PFS (11.5 mos. without radiation therapy, 88.1 mos. with postoperative radiation therapy, and >120 months with preoperative radiation therapy, *p* = 0.04). This is similar to Jones et al., who reported a local control of 95% for unplanned excision cases receiving preoperative radiation therapy followed by excision for highly selected cohort of patients [25]. Giuliano and Eilber also demonstrated 94.5% local control at 5 years for a similar cohort of patients [11].

In this study, PFS and LRFS were also impacted by patient performance status. This is revealing as perhaps patients who are offered multimodality therapy with preoperative radiation therapy and reexcision are healthier and thought to be able to tolerate the recommended treatments without increasing morbidity.

Limitations of this study include its retrospective nature and the consequent potential for patient heterogeneity and selection bias. Moreover, the sample size of the UE was relatively small and heterogeneous in nature with a relatively short follow-up. The minimum follow-up of patients in this study was 6 months (one patient), but the majority of patients have over 12-month follow-up, although this is still a follow-up period that may be short to evaluate local or distant disease and survival.

Evidence in the literature to support a treatment strategy after UE is limited and the treatment algorithm after UE at our institution was not well established. This allowed factors such as patient preference, convenience, and age to play disproportionate roles in the treatment strategy employed. Although it shows benefit to the use of preoperative radiation therapy followed by reexcision, it remains to be determined if all patients, regardless of their disease characteristics, may warrant this management. Additionally, in this study the methods of treatment in the UE group were heterogeneous and again there may be preselection bias in those that receive pre- or postoperative radiotherapy or reexcision alone. Also, only one patient received chemotherapy which could have benefits in terms of local and distant control. This, in the setting of a low sample size in UE group, should make the recommendations for additional therapy done on a case-by-case basis. Nevertheless, based on our study findings, our institutional treatment pathway will include preoperative XRT followed by wide surgical reexcision in hopes of minimizing the risk of LR and improving PFS.

## 5. Conclusions

The ideal treatment for sarcoma is with multimodal therapy in the setting of multidisciplinary tumor board. Despite aggressive education aimed at minimizing the incidence of unplanned excisions, a significant number of STS patients will continue to undergo UE by nonsarcoma specialists. Despite the limitation of our small sample size, our series has shown that preoperative radiation therapy followed by reexcision can improve local control and progression-free survival significantly, almost approaching that of patients who undergo planned STS excisions. Larger studies are needed to corroborate our findings. Our institutional recommendation for patients referred after unplanned soft tissue sarcoma excisions will be radiation therapy followed by wide reexcision of the operative bed.

## Figures and Tables

**Table 1:** Patient, disease, and treatment characteristics between patients who underwent UE and PE.

Variable	All patients	Planned excision	Unplanned excision
Number	245	211	34

Median age	57	57	64

Median tumor Size	7.9 cm	8.5 cm	4 cm

Stage	I: 46 (18.7%) II: 62 (25.3%) III: 137 (56%)	I: 40 (19%) II: 46 (22%) III: 125 (59%)	I: 6 (17.7%) II: 16 (47%) III: 12 (35.3%)

Grade	Low: 47 (19.1%) Intermediate: 20 (8.2%) High: 178 (72.7%)	Low: 45 (18.4%) Intermediate: 15 (6.1%) High: 185 (75.5%)	Low: (3%) Intermediate: 5 (14.7%) High: 28 (82.3%)

Histology	Undifferentiated: 56 (22.9%) Leiomyosarcoma/liposarcoma: 77 (31.4%) Synovial: 45 (18.4%) Other: 67 (27.3%)	Undifferentiated: 51 (24.1%) Leiomyosarcoma/liposarcoma: 68 (32.2%) Synovial: 38 (18%) Other: 48 (22.7%)	Undifferentiated: 6 (17.6%) Leiomyosarcoma/liposarcoma: 9 (26.5%) Synovial: 7 (20.6%) Other: 12 (35.3%)

Tumor location	Upper extremity: 64 (26.1%) Lower extremity: 181 (73.9%)	Upper extremity: 50 (23.7%) Lower extremity: 161 (76.3%)	Upper extremity: 13 (38.2%) Lower extremity: 21 (61.8%)

Subcutaneous disease (TXa)	45 (18.4%)	36 (17%)	9 (26.5%)

Timing of RT	No RT: 36 (14.7%) Preoperative: 174 (71%) Postoperative: 35 (14.3%)	No RT: 24 (11.4%) Preoperative: 163 (77.2%) Postoperative: 24 (11.4%)	No RT: 12 (35.4%) Preoperative: 11 (32.3%) Postoperative (alone or after reexcision): 11 (32.3%)

Chemotherapy	68 (28%)	67 (32%)	1 (3%)

**Table 2:** Post-unplanned excision therapies.

Intervention after UE	Patients (%)	Local recurrence (%)
*No radiation therapy*		
Chemotherapy alone	1 (2.9)	0/1 (0)
Reexcision alone	8 (23.5)	7/8 (87.5)
Observation alone	3 (8.8)	1/3 (33)

*Radiation therapy*		
RT alone	5 (14.7)	1/5 (20)
Reexcision followed by postoperative RT	6 (17.7)	2/6 (33)
Preoperative RT followed by reexcision	11 (32.4)	1/11 (9)

**Table 3:** Univariate analysis for local recurrence-free, progression-free, and overall survival (NS: not significant).

Variables	LRFS	PFS	OS
Age	NS	NS	NS
Tumor size	NS	NS	NS
Grade	NS	NS	NS
Stage	NS	NS	NS
Karnofsky performance status	*p* = 0.002	*p* = 0.0007	NS
Tumor location	NS	NS	NS
Chemotherapy	NS	NS	NS
Timing of RT	*p* = 0.06	*p* = 0.01	NS
